# Supplementary material for: A Novel Multi-Component Formulation Reduces Inflammation In Vitro and Clinically Lessens the Symptoms of Chronic Eczematous Skin
Source: Int J Mol Sci. 2023 Aug 19;24(16):12979. doi: 10.3390/ijms241612979 (PMC10454735; doi:10.3390/ijms241612979)
Supplement: Supplementary file 1 [file ijms-24-12979-s001.zip › ijms-2526314-supplementary.pdf]

SUPPLEMENTARY MATERIAL

1. Supplementary Materials and Methods

1.1 SPM

For *in vitro* cell culture experiments, culture media was supplemented with SPM components dissolved in Milli-Q water to generate 5x (or 500%) stocks and to obtain various concentrations while maintaining the same ratio of Milli-Q water added to the SPM-supplemented media.

For the clinical study, SPM components and a proprietary humectant formulation (Cosmax, South Korea) of 3% glycerin, 2% 1,2-hexanediol, 1.7% methyl propanediol, and 0.3% hydroxy acetophenone were dissolved in distilled water to generate a 1x (100%) SPM topical solution. The vehicle agent was identically generated without the addition of SPM components.

1.2 Cell viability assay

To determine the viability of HaCaT cells by MTT, HaCaT cells ($3.0x10^5$ cells/well) were plated in 24-well plates for 24 h. The cells were stimulated with 0 (control), 25, or 50 mg/mL of LPS for 24 h. After 24 h of incubation, cells were treated with 20% SPM for another 24 h. Subsequently, the media was replaced with MTT substrate diluted in DMEM (1:8, Cat # M6494, Thermo Fisher) and incubated at 37°C for 2 h, and the supernatants were then removed. 400 uL dimethyl sulfoxide (DMSO) was added to each well to dissolve the formazan crystals. The colored supernatants were transferred to a 96-well plate. The absorbance was measured at 540 nm.

1.3 RT-qPCR analysis

RNA was extracted using TRIzol Reagent (Ambion), chloroform, and isopropanol. RNA was reverse transcribed to cDNA using the PrimeScript RT Reagent Kit with gDNA Eraser (Takara). Real-time qPCR was performed with SensiFAST SYBR No-ROX Kit (Meridian) on a CFX96 Real-Time System (Bio-Rad). Target gene expression was quantified from the ratio of the target Cq (quantification cycle) values, calculated using CFX Manager software (Bio-Rad), to GAPDH Cq values from the same sample. The sequences of the primers are shown in **Supplementary Table 1**.

1.4 Automated time-course imaging using Incucyte

The viability of Hs27 cells was determined through automated time-course imaging using Incucyte (Sartorius.) Hs27 (5,000 cells/well) were seeded into 96-well plates and cultured for 24 h, followed by staining with 1:400 IncuCyte® NucLight Rapid Red (cat#4717, Sartorius), which labels nuclei, including daughter nuclei. The nuclei-labeled cells were allowed to grow in a dose range of LPS (50,100, and 150 μg/mL) while fluorescence and phase-contrast images were acquired every 1 h for 24 h. Results were normalized to the value observed at the beginning of the experiment. Fluorescent cells and nuclei were quantified using the IncuCyte® integrated analysis software.

1.5 Histological analysis

Skin biopsies were conducted on the SPM-applied lesion from one participant at the beginning and the end of the 3-week application. Skin samples were biopsied and fixed in a formalin solution (10%) for 24 h. Paraffin blocks were cut into 4.5-μm- thick sections. Sectioned samples were deparaffinized using a series of dilutions of xylene and ethanol at room temperature, then rinsed with deionized water. The sectioned samples were stained with hematoxylin and eosin and imaged by bright-field microscopy.

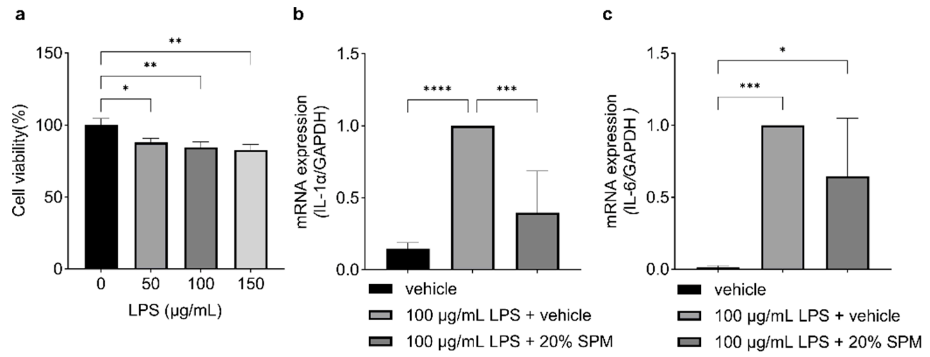

Figure S1. SPM suppresses LPS-induced IL-1a and IL-6 expression in Hs27 fibroblasts. (**a**) Hs27 cell viability after 24 h exposure to 0, 50, 100, or 150 mg/mL LPS measured by Incucyte. (**b**) Hs27 cells were stimulated with 100 mg/mL LPS for 24 h. After 24 h LPS treatment, LPS was removed, and cells were treated with 20% SPM or vehicle for another 24 h. RT-qPCR was used to determine mRNA expression levels. mRNA expression levels of IL-1α and (**c**) IL-6 were normalized to GAPDH mRNA expression level. Error bars represent the mean ± standard deviation. N=3 biological replicates. **p*<0.05; ***p*< 0.01; ****p*< 0.001; ****p< 0.0001, as determined by One-way ANOVA followed by Tukey’s multiple comparison test.

Table S1. Primers used in RT-PCR.

Primer	Type	Sequence (5’→3’)
GAPDH	Forward	ACC CAC TCC TCC ACC TTT GA
	Reverse	CTG TTG CTG TAG CCA AAT TCG T
IL-1β	Forward	AAG CTG ATG GCC CTA AAC AG
	Reverse	AGG TGC ATC GTG CAC ATA AG
IL-1α	Forward	AGA TGC CTG AGA TAC CCA AAA CC
	Reverse	CCA GCT ATG AAC TCC TTC TC
IL-6	Forward	CCA GCT ATG AAC TCC TTC TC
	Reverse	GCT TGT TCC TCA CAT CTC TC
